# Adherence to Perioperative Antibiotic Prophylaxis Recommendations and Its Impact on Postoperative Surgical Site Infections

**DOI:** 10.7759/cureus.25859

**Published:** 2022-06-11

**Authors:** Claudia Berrondo, Marco Carone, Cindy Katz, Avi Kenny

**Affiliations:** 1 Surgery/Pediatric Urology, University of Nebraska Medical Center, Omaha, USA; 2 Pediatric Urology, Children’s Hospital and Medical Center, Omaha, USA; 3 Biostatistics, University of Washington, Seattle, USA; 4 Surgery/Surgical Quality Improvement, Seattle Children's Hospital, Seattle, USA

**Keywords:** infection prevention, nsqip peds, american college of surgeons, peri-operative antibiotic prophylaxis, surgical site infection

## Abstract

Introduction

Surgical site infections (SSIs) are common and carry a significant risk of morbidity and mortality and lead to increased healthcare costs. Perioperative antibiotic prophylaxis decreases the risk of SSIs. There are several guidelines on the use of perioperative antibiotic prophylaxis. The American College of Surgeons (ACS) recommends weight-based antibiotic administration within 60 minutes prior to (two hours for vancomycin/fluoroquinolones) incision and redosing by drug half-life. There are limited data regarding adherence to existing recommendations. Furthermore, there are scarce data on the relationship between adherence to recommendations and the risk of postoperative SSI.

Objectives

In this study, we aimed to assess the adherence to ACS guidelines for perioperative antimicrobial prophylaxis in the Seattle Children’s Hospital (SCH) National Surgical Quality Improvement Program (NSQIP) pediatric cohort and to determine whether adherence to ACS guidelines is associated with a decreased risk of SSI. the secondary objective was to identify risk factors associated with SSI in our patient population.

Materials and methods

We conducted a secondary analysis of an institutional NSQIP pediatric data cohort between Jan 1, 2012, and Dec 31, 2017. We calculated summary statistics to assess adherence to ACS recommendations and fit a logistic regression model to identify factors associated with the risk of SSI. Patients who did not receive antibiotic prophylaxis were excluded.

Results

A total of 6,072 surgeries among 5,532 patients met the inclusion criteria. Adherence was achieved for weight-based dosing in 35% of surgeries, administration prior to the incision in 91%, administration within 60 minutes (two hours for vancomycin/fluoroquinolones) in 86%, correct redosing in 97%, and to all recommendations in 29%. There were no significant associations between any adherence metrics and SSI, although confidence intervals were wide for some metrics. Factors associated with SSI when adherence was met included urgent case status, wound class 2 or 4, the American Society of Anesthesiologists (ASA) class 2-5, and surgery duration.

Conclusion

There was varying adherence to ACS recommendations on antibiotic prophylaxis in our cohort. More evidence is needed to better understand the effects of adherence to any or all components of the recommendations on SSI. We identified a group of pediatric patients at risk of SSI and a need for further research and targeted interventions.

## Introduction

Surgical site infections (SSI) are one of the most common nosocomial infections and account for significant morbidity, mortality, and healthcare costs [1-3]. The use of perioperative antibiotics has been shown to help decrease the risk of developing a postoperative SSI in many surgical disciplines [4-6]. However, there is increasing evidence that inappropriate antibiotic use is associated with antibiotic-related complications such as adverse reactions, the emergence of resistant organisms, and Clostridioides difficile infection [7-10].

Recommendations and guidelines for the type and duration of prophylaxis have been provided by several organizations with some variability in the indications for prophylaxis and the ideal agent between organizations. The general consensus in terms of the timing and duration of antibiotic prophylaxis among different organizations is that one-time intraoperative dosing within 60 minutes prior to (or two hours for vancomycin of fluoroquinolones) of the surgical incision (with or without intraoperative redosing for long-duration procedures, excessive blood loss, etc.) is sufficient, but that up to 24 hours of antibiotics is acceptable. The most recent publications from the Centers for Disease Control and Prevention (CDC), World Health Organization (WHO), and American College of Surgeons (ACS) recommend against administering antibiotics after skin closure in clean and clean-contaminated procedures, even with the presence of a postoperative drain [11-13]. Although there is a consensus regarding the timing and duration of prophylactic antibiotics, as well as dosing for the antibiotics used, there is significant variation among different society recommendations for both adult and pediatric patients as to which procedures warrant prophylaxis and which antibiotic is recommended. There are also limited data on adherence to different components of existing guidelines (timing of administration of the antibiotic, appropriate antibiotic dosing, and appropriate antibiotic redosing). Furthermore, there are scarce data on the relationship between adherence to guidelines (and/or components of the guidelines) and the risk of SSI.

In this study, our primary objectives were to assess adherence to the ACS guidelines for perioperative antimicrobial prophylaxis in the Seattle Children’s Hospital (SCH) National Surgical Quality Improvement Program (NSQIP) pediatric data cohort and to determine whether adherence to ACS guidelines is associated with a decreased risk of SSI. The secondary objective was to identify risk factors associated with SSI in the SCH patient population.

## Materials and methods

Study design

This study received ethical approval from the SCH Institutional Review Board (STUDY00001702). We conducted a retrospective cohort study of the SCH NSQIP Pediatric (Peds) database cohort between January 1, 2012, and December 31, 2017. Once the eligible patients were identified, information was collected, de-identified, and stored in a password-protected file. Patients who did not receive intraoperative antibiotics or those with missing data regarding intraoperative antibiotics were excluded.

Antibiotic prophylaxis guidelines

Antibiotic prophylaxis guidelines were determined according to the ACS guidelines on SSI prevention updated in 2016. There were no pertinent differences between prior guidelines and the 2016 update. The recommendations included (1) antibiotic administration prior to incision, (2) weight-based antibiotic dosing (Table 4), (3) antibiotic administration within 60 minutes prior to incision (or 120 minutes for vancomycin or fluoroquinolones), and (4) antibiotic redosing when appropriate (Table 4) [12].

Demographic and clinical data were extracted from the SCH NSQIP Peds database and included age, sex, race, ethnicity, weight, medical comorbidities, surgical specialty, date, and time of surgery. Data on prophylactic antibiotic administration including type, dose, and timing were extracted from the electronic medical records.

Surgical site infections

The primary outcome was SSI within 30 days after surgery, as defined by NSQIP Peds. NSQIP Peds records 30-day postoperative outcomes (including SSIs) on a sample of operations performed at participating hospitals as previously described. Surgical procedures included in NSQIP Peds were identified using a list of Current Procedural Terminology (CPT) codes defined by NSQIP and updated yearly [14,15]. For the purposes of this study, NSQIP-defined superficial incisional SSI, deep incisional SSI, and organ space SSI were all combined into a single composite outcome of SSI.

Statistical analysis

Summary statistics were used to assess adherence to ACS recommendations. For each ACS recommendation, the existence of an association between SSI after surgical procedure and adherence to this recommendation was evaluated using a chi-square test of independence. The existence of an association between SSI after surgical procedure and adherence to all ACS recommendations was evaluated in a similar fashion. To assess the association between surgical procedures and adherence to recommendations while controlling for potential confounding factors [by age, sex, race, ethnicity, weight on the day of surgery, surgical subspecialty, type of admission, case status (elective, urgent, or emergent), wound classification, the American Society of Anesthesiologists (ASA) classification, and surgery duration], and to identify factors associated with the risk of SSI, we used a logistic regression model fit via generalized estimating equations to account for correlation across procedures performed in the same patient. All statistical analyses were performed using R Studio (Version 1.2.1335; R Foundation for Statistical Computing, Vienna, Austria), and all statistical tests were conducted using a significance level of α=0.05.

## Results

Of the 7,182 surgeries in the SCH NSQIP Peds database spanning the five-year time period identified, 6,072 (84.5%) had data available on perioperative antibiotic use and were therefore included in the analysis. These surgeries were performed on a total of 5,532 patients. Adherence to ACS recommendations was met for weight-based dosing in 35% of surgeries, for administration prior to the incision in 91% of surgeries, for the timing of administration of antibiotics prior to the incision in 86% of surgeries, and for antibiotic redosing in 97% of surgeries. Adherence to all ACS recommendations was achieved in 29% of surgeries (Table 1).

**Table 1:** Characteristics of the study population* *Summarized at the level of surgery, the unit of analysis Q1 – Q3: interquartile range, ACS: The American College of Surgeons

Characteristic		Values
Age, years, median (Q1 – Q3)		7.1 (1.3 – 12.4)
Sex, % (n)	Male	54.9 (3,336)
Race, % (n)	American Indian or Alaska Native	2.6 (157)
	Asian	8.7 (528)
	Black or African American	5.1 (309)
	Native Hawaiian or other Pacific Islander	1.4 (88)
	Unknown/not reported	23.3 (1,412)
	White	58.9 (3,578)
Ethnicity, % (n)	Hispanic	19.4 (1,179)
Height, cm, median (Q1 – Q3)		115 (76 – 149)
Weight, kg, median (Q1 – Q3)		22.9 (10.2 – 44.5)
Type of admission, % (n)	Inpatient	64.5 (3,916)
	Outpatient	35.5 (2,156)
ACS recommendation, % (n)	Correct weight-based dosing	34.6 (2,093)
	Administration prior to incision	91.1 (5,530)
	Correct timing of administration	85.5 (5,193)
	Correct redosing	97.4 (5,913)
	All recommendations	28.5 (1,726)

In both univariable and multivariable analyses, there were no significant associations between adherence to ACS recommendations and SSI (Table 2).

Factors associated with SSI when adherence was controlled for included urgent case status, surgical wound classifications 2 and 4, ASA class 2-5, and surgery duration (Table 3).

**Table 2:** Association between adherence to the American College of Surgeons (ACS) antibiotic prophylaxis recommendations and surgical site infections (SSIs) The univariable associations are the percentage of each adherence group that experienced an SSI. The multivariable associations are odds ratios of the association between adherence and SSI, controlling for potential confounders, obtained from a logistic regression model SSI: surgical site infection

Recommendation		Univariable associations			Multivariable associations	
		% SSI (n / total)	95% CI	P-value	Odds ratio (95% CI)	P-value
Weight-based dosing	Adherent	2.4 (51 / 2,093)	1.8 – 3.1	0.33	0.75	0.26
	Non-adherent	2.0 (80 / 3,961)	1.6 – 2.5		(0.45 – 1.25)	
Administration prior to incision	Adherent	2.2 (122 / 5,530)	1.8 – 2.6	0.49	1.79	0.25
	Non-adherent	1.7 (9 / 542)	0.6 – 2.7		(0.66 – 4.80)	
Timing of administration	Adherent	2.2 (112 / 5,193)	1.8 – 2.6	1.00	1.12	0.77
	Non-adherent	2.2 (19 / 879)	1.2 – 3.1		(0.51 – 2.46)	
Redosing	Adherent	2.1 (124 / 5,913)	1.7 – 2.5	0.09	0.84	0.70
	Non-adherent	4.4 (7 / 159)	1.2 – 7.6		(0.34 – 2.08)	
All recommendations	Adherent	2.4 (42 / 1,726)	1.7 – 3.2	0.42	0.97	0.88
	Non-adherent	2.1 (89 / 4,328)	1.6 – 2.5		(0.60 – 1.55)	

**Table 3:** Risk factors associated with SSI* *Assessed via a multivariable logistic regression analysis controlling for adherence to ACS recommendations ACS: The American College of Surgeons; SSI: surgical site infection; ASA: The American Society of Anesthesiologists

Variable		Odds ratio (95% CI)	P-value
Age, years		0.99 (0.94 – 1.04)	0.65
Sex	Male	Reference	0.74
	Female	0.94 (0.66 – 1.35)	
Race	American Indian or Alaska Native	Reference	
	Asian	1.87 (0.45 – 7.81)	0.39
	Black or African American	0.44 (0.06 – 3.15)	0.42
	Native Hawaiian or other Pacific Islander	2.69 (0.49 – 14.7)	0.25
	Unknown/not reported	2.42 (0.61 – 9.61)	0.21
	White	1.98 (0.53 – 7.44)	0.31
Ethnicity	Not Hispanic	Reference	
	Hispanic	0.84 (0.49 – 1.43)	0.52
	Unknown	0.23 (0.05 – 1.10)	0.07
Weight, kg		1.01 (0.996 – 1.02)	0.24
Surgical specialty	Neurosurgery	Reference	
	Orthopedic surgery	0.68 (0.33 – 1.40)	0.29
	Otolaryngology	1.26 (0.54 – 2.89)	0.59
	Plastic surgery	0.80 (0.32 – 2.01)	0.63
	General surgery	0.69 (0.34 – 1.40)	0.31
	Urology	0.46 (0.16 – 1.32)	0.15
Type of admission	Inpatient	Reference	
	Outpatient	0.89 (0.50 – 1.58)	0.69
Case status	Elective	Reference	
	Urgent	1.89 (1.12 – 3.20)	0.01
	Emergent	1.24 (0.64 – 2.40)	0.53
Wound classification	Clean	Reference	
	Clean/contaminated	1.93 (1.05 – 3.54)	0.03
	Contaminated	1.24 (0.36 – 4.24)	0.73
	Dirty/infected	3.43 (1.49 – 7.89)	0.004
ASA classification	1	Reference	
	2	2.23 (1.18 – 4.22)	0.01
	3	4.15 (2.15 – 8.02)	<0.001
	4	5.79 (2.54 – 13.2)	<0.001
	5	N/A	N/A
	None assigned	18.0 (0.78 – 412)	0.07
Surgery duration, minutes		1.27 (1.16 – 1.38)	<0.001

## Discussion

SSIs are one of the most common and costly postoperative complications, and the use of perioperative antibiotics has been shown to decrease the risk of SSI in many surgical disciplines [1-6]. We found poor adherence to current ACS recommendations in the SCH NSQIP cohort of children who underwent surgery at our institution. In addition, we did not find any significant associations between compliance with ACS antimicrobial prophylaxis recommendations and incidence of SSI in patients who received antibiotic prophylaxis, although the associated confidence intervals were wide. We did find factors associated with SSI in patients where adherence was met, including urgent case status, wound classifications 2 and 4, ASA class 2-5, and surgery duration.

To our knowledge, this is the first study to evaluate the impact of adherence to ACS recommendations for surgical antimicrobial prophylaxis (including timing, dosing, and redosing) and the development of SSI in pediatric patients. Mohamed Rizvi et al. reported low adherence to guidelines for antimicrobial surgical prophylaxis in pediatric patients (39.6%) but did not evaluate the association between adherence and the development of SSI [16]. Bardia et al. found poor adherence to guidelines (overall 35.9%, 19.7% for antibiotic choice, 17.1% for weight-adjusted dosing, 0.6% for timing of the first dose, and 26.8% for redosing) [17]. Other similar studies have demonstrated similar results with poor adherence to recommendations on antibiotic prophylaxis [18-20].

Previous studies investigating the relationship between antimicrobial prophylaxis and SSI have yielded conflicting results. We did not find an association between weight-based antibiotic dosing and SSI in our patient population, but this may be due to the large statistical uncertainty resulting from the low rate of SSIs in this population. To our knowledge, there are no published studies investigating this relationship.

Hawn et al. found no association between the timing of antibiotic prophylaxis and the development of SSI in adult patients who underwent hip or knee arthroplasty [21]. Litz et al. found no association between the timing of antibiotic prophylaxis and SSI in pediatric patients undergoing appendectomy [22]. In a systematic review, de Jonge et al. found an increased risk of SSI in patients who received antibiotics >120 minutes prior to the incision, or after the incision [23]. Toor et al. found a decrease in SSIs after the intervention to improve the appropriate timing of administration of preoperative antimicrobial prophylaxis in patients at least 12 years of age undergoing non-cardiac, non-obstetric surgery [24]. Chandrananth et al. found that non-adherence to antimicrobial prophylaxis increased the risk of SSI in adult patients undergoing total knee and hip arthroplasty [25]. Khoshbin et al. found a decrease in SSIs in pediatric patients who received antibiotic prophylaxis within 60 minutes prior to surgical incision [26].

Although we did not identify any relationship between appropriate intraoperative antibiotics redosing and SSI in the SCH patient population, similar previously published studies in adult patients have demonstrated some benefits. Zanetti et al. have demonstrated that redosing of antibiotics decreased the risk of SSI after cardiac surgery if the duration of surgery was >400 minutes [27]. Similarly, Kasatpibal et al. demonstrated that intraoperative antimicrobial redosing during long operations decreased the risk of SSI in multiple types of surgical procedures in adult patients [28]. Bertschi et al. also found that intraoperative antibiotic redosing in prolonged surgical cases >240 minutes decreased the risk of SSI regardless of the exact timing of redosing in adult patients undergoing abdominal, vascular, or trauma surgery [29].

In the SCH patient population, we found that urgent case status, wound classifications of 2 and 4, ASA class of 2-5, and longer surgery duration were associated with an increased risk of SSI in patients where adherence to recommendations was met. Prospero et al. found that adherence to an antibiotic prophylaxis protocol decreased the risk of SSI in adult patients, and they also identified several risk factors associated with SSI including ASA score 2-5, wound class 2-4, longer duration of surgery, and urgent surgery [30]. Additionally, Kasatpibal et al. found that improper antibiotic redosing, inpatient status, smoking, emergency surgery, bowel surgery and pancreatectomy, intraoperative blood transfusion >500 mL, and multiple procedures all increased the risk of SSI [28]. Bertschi et al. found that surgery duration >240 minutes was associated with an increased risk of SSI [29].

There are several limitations to our study. The data we studied included patients from a single institution and were retrospectively collected, and hence our results may not be generalizable. There may also be unmeasured confounding variables, such as intraoperative temperature, intraoperative glucose, preoperative or intraoperative skin preparation, obesity, and diabetes mellitus, which were not accounted for in our analysis. In addition, the cohort studied consisted of patients captured by the SCH NSQIP Peds database, which includes only a sampling of patients undergoing a defined set of surgical procedures. As a result, the sample is not necessarily representative of all pediatric patients undergoing surgical intervention. As there are no current guidelines from ACS as to which patients should receive antibiotics, we also excluded patients who did not receive perioperative antibiotic prophylaxis. Excluding this group of patients likely introduced a selection bias that cannot be accounted for in this study. Furthermore, since existing guidelines on the need for antibiotic prophylaxis and choice of antibiotic prophylaxis are extremely limited in the pediatric patient population, we did not evaluate the appropriateness of administration of antibiotics or choice of antibiotics.

## Conclusions

Based on our findings, adherence to current perioperative antibiotic prophylaxis guidelines is limited. In the SCH NSQIP Peds cohort of patients who received perioperative antibiotic prophylaxis, we did not find significant associations between adherence to ACS guidelines and the development of SSIs. We found that urgent cases status, wound classifications of 2 and 4, ASA class of 2-5, and longer surgery duration were associated with an increased risk of SSI in this patient population. Further research is needed to better understand the relationship between adherence to existing antibiotic prophylaxis guidelines and SSIs, especially in pediatric patients.

## Appendices

Table 4 lays out the details on weight-based dosing and redosing intervals for antibiotic prophylaxis.

**Table 4:** Weight-based dosing and redosing intervals for antibiotic prophylaxis

Antimicrobial	Dose	Redosing interval (hours)
Ampicillin-sulbactam	50 mg/kg of the ampicillin component	2
Ampicillin	50 mg/kg	2
Aztreonam	30 mg/kg	2
Cefazolin	30 mg/kg	4
Cefuroxime	50 mg/kg	4
Cefotaxime	50 mg/kg	3
Cefoxitin	40 mg/kg	2
Cefotetan	40 mg/kg	6
Ceftriaxone	50-75 mg/kg	NA
Ciprofloxacin	10 mg/kg	NA
Clindamycin	10 mg/kg	6
Ertapenem	15 mg/kg	NA
Fluconazole	6 mg/kg	NA
Gentamicin	2.5 mg/kg	NA
Levofloxacin	10 mg/kg Neonates <1200 g 7.5 mg/kg	NA
Metronidazole	15 mg/kg	NA
Moxifloxacin	10 mg/kg	NA
Piperacillin-tazobactam	2-9 mo 80 mg/kg of the piperacillin component >9 months and <40 kg 100 mg/kg of piperacillin component	2
Vancomycin	15 mg/kg	NA
